# Effect of Selective Muscle Training Using Visual EMG Biofeedback on Infraspinatus and Posterior Deltoid

**DOI:** 10.2478/hukin-2014-0113

**Published:** 2014-12-30

**Authors:** One-bin Lim, Jeong-ah Kim, Si-jeong Song, Heon-seock Cynn, Chung-hwi Yi

**Affiliations:** 1 Department of Physical Therapy, The Graduate School, Yonsei University, Won-ju, Republic of Korea.; 2 Department of Physical Therapy, Seoul-chuk Hospital, Seoul, Republic of Korea.; 3 Department of Physical Therapy, College of Health Science, Yonsei University, Won-ju, Republic of Korea.

**Keywords:** EMG biofeedback, Infraspinatus, Posterior deltoid, Shoulder external rotation

## Abstract

We investigated the effects of visual electromyography (EMG) biofeedback during side-lying shoulder external rotation exercise on the EMG amplitude for the posterior deltoid, infraspinatus, and infraspinatus/posterior deltoid EMG activity ratio. Thirty-one asymptomatic subjects were included. Subjects performed side-lying shoulder external rotation exercise with and without visual EMG biofeedback. Surface EMG was used to collect data from the posterior deltoid and infraspinatus muscles. The visual EMG biofeedback applied the pre-established threshold to prevent excessive posterior deltoid muscle contraction. A paired t-test was used to determine the significance of the measurements between without vs. with visual EMG biofeedback. Posterior deltoid activity significantly decreased while infraspinatus activity and the infraspinatus/posterior activity ratio significantly increased during side-lying shoulder external rotation exercise with visual EMG biofeedback. This suggests that using visual EMG biofeedback during shoulder external rotation exercise is a clinically effective training method for reducing posterior deltoid activity and increasing infraspinatus activity.

## Introduction

Shoulder pain is a common musculoskeletal problem. Reported incidences of shoulder pain are 11.2–29.5 per 1000 person-years (Bot et al., 2005; Linsell et al., 2006) and the prevalence is 4.7–46.7 per 1000 person-years (Linsell et al., 2006; Luime et al., 2004; Picavet and Schouten, 2003). Shoulder pain often has a long course with recurrence (Reilingh et al., 2008). The high degree of mobility of the shoulder joint enables the hand to perform various activities (Dark et al., 2007; Tardo et al., 2013; Wuelker et al., 1998). The shoulder joint depends on dynamic support from surrounding muscles to achieve functional stability without compromising mobility (Dark et al., 2007; Pagnani and Warren, 1994; Tardo et al., 2013). Rotator cuff muscles (supraspinatus, infraspinatus, subscapularis, and teres minor) have a dynamic stabilizing function at the shoulder joint (Tardo et al., 2013; Wuelker et al., 1998).

Normal movement depends on co-contraction of rotator cuff muscles to provide stabilization of the humeral head in the concave glenoid fossa (Bitter et al., 2007; David et al., 2000). In the coronal plane, inferior translation and compressive forces produced by the infraspinatus, subscapularis, and teres minor counterbalance the superior pull of the deltoid and supraspinatus (Bitter et al., 2007). In the transverse plane, balance of the infraspinatus and subscapularis controls anteroposterior translation of the humeral head (Bitter et al., 2007; Burkhart, 1991). This co-activation limits superior humeral head translation (Bitter et al., 2007; Friedman and Knetsche, 1996). An over-activated posterior deltoid with a less-activated infraspinatus contributes to increased anterior humeral gliding (Caldwell et al., 2007; Sharmann, 2002). The rotator cuff plays an important role in prevention of unwanted humeral head migration and impingement on subacromial structures (Bitter et al., 2007).

Rotator cuff retraining programs restore the balance between the deltoid, which elevates the humeral head, and the rotator cuff (Haahr et al., 2005). Physical therapy interventions include shoulder external rotation exercises performed while maintaining adduction to increase stabilization and strength of the infraspinatus (Haahr et al., 2005). Shoulder adduction isolates the infraspinatus from the posterior deltoid, and the side-lying external rotation exercise may be the most effective maneuver to increase infraspinatus activation (Reinold et al., 2004). In the present study, a side-lying shoulder external rotation exercise was used to activate the infraspinatus and reduce the activity of the posterior deltoid.

Electromyography (EMG) biofeedback is a learning tool recommended for motor control in rehabilitation (Basmajian, 1981; Holermann et al., 2009; Holermann et al., 2010). Visual EMG biofeedback training uses an electronic device to display instantaneous information on physiological events, which allows the user to control involuntary muscle contraction by manipulating the monitor signals (Basmajian, 1981; Holermann et al., 2009; Holermann et al., 2010). This modality allows subjects to learn how to have fine control over the activity of any given target muscle (Basmajian, 1981; Holermann et al., 2009; Holtermann et al., 2010). Visual EMG biofeedback training has been used in rehabilitation programs to learn selective muscle contraction (Holtermann et al., 2009; Holtermann et al., 2010; Huang et al., 2013). However, no previous study has investigated training with this modality to decrease activation of the posterior deltoid while increasing activation of the infraspinatus. In the present study, we determined the effect of visual EMG biofeedback during side-lying shoulder external rotation exercise on posterior deltoid and infraspinatus activation and the infraspinatus/posterior deltoid EMG activity ratio. We hypothesized that side lying shoulder external rotation exercise with biofeedback would reduce the posterior deltoid activity but increase both the activity of the infraspinatus and the activity ratio.

## Material and Methods

### Participants

*A priori* power analysis with G*Power software (G*Power ver. 3.1.5, Franz Faul, University of Kiel, Germany) was used to estimate the sample size. A pilot study with six asymptomatic subjects was used to achieve an effect size of .63, an alpha level of 5%, and power of 80%. The estimated sample size was 17. Thirty-one asymptomatic subjects (21 males and 10 females) participated in the study. Normal shoulder function was defined as lack of shoulder pain for 2 years and no medical or physical therapy treatment for shoulder pain. Subjects were required to demonstrate a normal range of motion of the shoulder and scapulohumeral rhythm (assessed visually by two experienced physical therapists). Subjects also had to be pain-free during maximal voluntary isometric internal and external rotation strength tests (Tardo et al., 2013). Exclusion criteria were as follows: (1) history of shoulder joint surgery, (2) fracture of the shoulder complex, (3) cervical problems, and (4) glenohumeral instability (Huang et al., 2013). Each subject signed an informed consent form and the rights of each subject were protected. The research protocol was approved by the Yonsei University Wonju Campus Human Studies Committee. The general characteristics of subjects are presented in Table 1.

### Instruments

#### Surface EMG

A Noraxon TeleMyo DTS Telemetry system (Noraxon Inc., Scottsdale, AZ, USA) was used to collect electromyographic signals from the posterior deltoid and infraspinatus. The electrode sites were shaved and cleaned with rubbing alcohol. Surface electrode pairs were positioned at an inter-electrode distance of 2 cm. Reference electrodes were integrated on backside of the DTS transmission probe. EMG data were collected for the posterior deltoid (electrodes were placed 2 cm below the lateral border of the spine of the scapula and angled obliquely to the arm) and the infraspinatus (electrodes were parallel to and approximately 4 cm below the spine of the scapula on the lateral aspect over the infrascapular fossa) (Criswell, 2011). EMG signals were amplified, band-pass filtered (10 and 450 Hz), and notch filtered (60 Hz, 120 Hz) before being digitally recorded at 1000 Hz and processed into the root mean square (RMS) with a moving window (1 s duration and 100 ms steps) throughout the entire recording.

#### Visual biofeedback device

**Figure 1:**
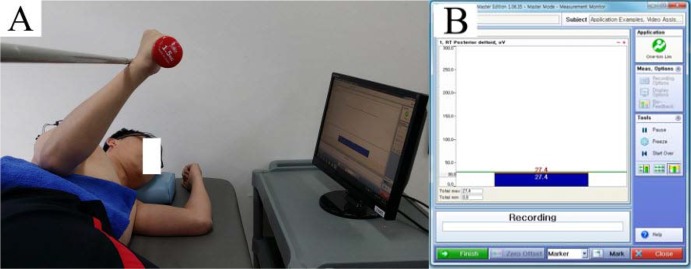
Side-lying shoulder external rotation exercise using visual EMG biofeedback: A: Shoulder external rotation of 45°; B: Visual EMG biofeedback monitor display.

The visual biofeedback device was used with the biofeedback measurement option in the Noraxon MyoResearch Master Edition 1.08 XP software (Noraxon, Inc., Scottsdale, AZ, USA). The biofeedback applied the pre-established threshold to prevent excessive posterior deltoid muscle contraction and give visual feedback. The threshold was calculated by averaging three MVIC values of the posterior deltoid for each subject. The EMG amplitude threshold for reducing the posterior deltoid muscle amplitude was determined based on 10% variation in the maximal voluntary isometric contraction (MVIC) (Jeon et al., 2011; Martins et al., 2008). This was displayed on a computer screen during shoulder external rotation exercise. The display’s vertical axis was EMG amplitude. The green horizontal bar was the pre-established threshold bar. The color of the amplitude bar changed to blue when the amplitude was below 10% of the MVIC, and changed to red when the value was above 10% of the MVIC (Jeon et al., 2011) (Figure 1).

### Procedures

The dominant shoulder, belonging to the extremity used for writing and eating, was used for testing. All subjects were right-arm dominant. Subjects laid on their side with the shoulder in a neutral position and the elbow in 90° flexion. From the starting position with the forearm resting on the abdomen, subjects moved the tested arm to a position of external rotation (45°) until the dorsal forearm touched the target bar and maintained the shoulder in 45° external rotation (Ha et al., 2013) (Figure 1).

Subjects performed shoulder external rotation with a towel between the trunk and elbow to avoid compensatory movements and enhance stability (Huang et al., 2013). A 45° external rotation of the shoulder was performed without and with visual EMG biofeedback. Biofeedback consisted of rising bars displayed on a computer screen corresponding to the muscle of interest. The bars presented the signal amplitude in an easy-to-read display and showed the threshold ranges to provide a target for EMG activation. The subjects were instructed to monitor the amplitude bar color and be sure not to exceed the threshold of the posterior deltoid while performing the exercise. The subjects received feedback of the amplitude to keep it as low as possible. The study participants were allowed to familiarize themselves with the standard position and movements for 10 min before data collection (Jeon et al., 2011). After the practice session, data were recorded for three trials of each exercise. Each subject performed the exercise without biofeedback followed by exercise with visual EMG biofeedback. They were given 8 s to complete one movement cycle (initial 3 s spent moving to the target position; last 5 s holding the target position). A metronome was used to control the speed and duration of movement. A beeping sound produced by the Noraxon MyoResearch Master Edition 1.08 XP software (Noraxon, Inc., Scottsdale, AZ, USA) was used as the start signal. To increase the EMG activity of the posterior deltoid and infraspinatus, subjects held a 1.5 kg load during the exercise.

### Data collation

Data on the EMG activity of the posterior deltoid and infraspinatus muscles were collected during the exercise. The mean EMG activity data obtained during the middle 3 s of the 5-s period was used for data analysis. The EMG data were normalized by calculating the mean RMS of three trials for the MVIC of each muscle. Posterior deltoid muscle activity was tested in a prone position with the arm abducted 90° and in neutral rotation (palm down). Resistance was applied in an anterior direction just proximal to the elbow (Kendall et al., 2005). Infraspinatus muscle activity was tested in the sitting position with the shoulder at 0° abduction, neutral rotation, and the elbow flexed to 90°. Resistance was applied just above the wrist to create internal shoulder rotation (Magee, 2007). Each contraction was held for 5 s with maximal effort against manual resistance. The first and last second of EMG data from each MVIC trial were discarded using the remaining 3 s of data. The mean MVIC value for the three trials was calculated. Data for each trial were expressed as a percentage of the calculated mean RMS of the MVIC (%MVIC). The mean %MVIC of the three trials and activity ratio were used in the analyses. Before taking the average for the final analysis, we used the intraclass correlation coefficient (ICC 3,1) to calculate the reliability of posterior deltoid and infraspinatus muscles in the without biofeedback condition and found excellent intra–rater reliability (ICC = 0.88 and 0.87, respectively).

### Statistical analysis

Descriptive statistics were calculated for all variables. The one-sample Kolmogorov–Smirnov test was performed to determine whether continuous data approximated a normal distribution. The paired t-test was used to compare the EMG amplitude and the activity ratio during shoulder external rotation under two different experimental conditions (within factor: without and with visual EMG biofeedback). The level of significance was set at α=.05. Analyses were performed using the SPSS ver. 21.0 software (SPSS, Inc., Chicago, IL, USA).

## Results

### Posterior deltoid

**Figure 2:**
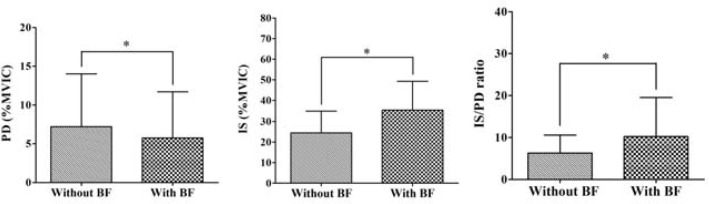
Comparison of the EMG activity during shoulder external rotation exercise without and with BF (PD: posterior deltoid; IS: infraspinatus; IS/PD ratio: infraspinatus/posterior deltoid ratio; BF: biofeedback; %MVIC: % maximal voluntary isometric contraction; error bars: standard deviation; *p<.05).

Figure 2 shows the results of posterior deltoid activity during the exercise without and with visual EMG biofeedback. Activity significantly decreased with visual EMG biofeedback (7.21±6.82 %MVIC without biofeedback; 5.75±5.95 %MVIC with biofeedback; t=3.62; p=.001).

### Infraspinatus

Figure 2 shows the results of infraspinatus activity during the exercise without and with visual EMG biofeedback. The activity significantly increased with biofeedback (27.47±10.55 %MVIC without biofeedback; 35.50±13.76 %MVIC with biofeedback; t=−5.30; p<.001).

### Infraspinatus/posterior deltoid activity ratio

Figure 2 shows the results for the activity ratio, which significantly increased with biofeedback (6.31±4.26 without, 10.23±9.30 with biofeedback; t=−3.65; p=.001).

## Discussion

This is the first study to investigate the immediate effects of visual EMG biofeedback training on the ability to decrease activation of the posterior deltoid and increase the activation of infraspinatus in asymptomatic subjects during the side-lying shoulder external rotation exercise. Previous studies have used EMG activities between shoulder muscle groups of interest to demonstrate the relative control of shoulder external rotation (Bitter et al., 2007; Ha et al., 2013; Reinold et al., 2004; Reinold et al., 2009).

We found significantly lower posterior deltoid activity during the shoulder external rotation exercise while using visual EMG biofeedback. Such biofeedback training has been shown to be effective for developing selective muscle control (Holtermann et al., 2009; Holtermann et al., 2010; Huang et al., 2013). Also, surface EMG signals change in the presence of altered afferent information required to sustain force (Farina et al., 2008). Visual EMG biofeedback training specifically targets insufficient muscle activation and can alter motor control and abnormal muscle activation patterns (Holtermann et al., 2009; Holtermann et al., 2010). Our results support previous studies that have suggested that biofeedback training may be clinically helpful for selectively reducing activation of the posterior deltoid and increasing recruitment of the infraspinatus during the side-lying shoulder external rotation exercise.

Infraspinatus activity significantly increased during the exercise with visual EMG biofeedback. Increased infraspinatus EMG activity may be associated with reduced activation of the posterior deltoid. In a previous study, patients with shoulder impingement syndrome or infraspinatus weakness that performed this exercise had excessive posterior deltoid muscle contraction compared to infraspinatus muscle contraction (Grimsby et al., 2009). To inhibit such excess contraction, many authors advocate selective activation of the infraspinatus muscle during shoulder external rotation exercises (Bitter et al., 2007; Ha et al., 2013; Reinold et al., 2004; Reinold et al., 2009). Unwanted anterior glide of the humeral head during shoulder external rotation is the result of the posterior deltoid becoming the dominant external rotator (Caldwell et al., 2007; Sharmann, 2002). Sahrmann (2002) suggested that for rotator cuff muscle exercises to be effective, the infraspinatus and teres minor muscles must optimally participate, and the posterior deltoid muscle should not be the primary rotator. Because posterior deltoid activity was reduced by adding visual EMG biofeedback, it follows that more infraspinatus activation was required to perform the same exercise.

This study supports the hypothesis that use of visual EMG biofeedback increases the infraspinatus/posterior deltoid EMG activity ratio during the shoulder external rotation exercise. The higher ratio signifies that the posterior deltoid was deactivated while the infraspinatus was highly activated. Higher ratios are of concern when choosing infraspinatus activation exercises, so performing exercise with visual EMG biofeedback is beneficial. Physical therapy interventions for rotator cuff retraining generally focus on restoration of functionality by utilizing target muscles with minimal participation of surrounding musculature (Reinold et al., 2009). An exercise with low posterior deltoid activity would facilitate selective activation of the infraspinatus muscle and reduce the risk of shoulder joint instability in subjects with shoulder impingement syndrome or infraspinatus weakness. Clinically, the rotation exercise with biofeedback can increase the infraspinatus/posterior deltoid EMG activity ratio by reducing posterior deltoid EMG activity and increasing infraspinatus EMG activity.

The practical implications for coaches and athletes are as follows. Our finding indicates that side-lying shoulder external rotation exercise with biofeedback is a better choice for selectively strengthening the infraspinatus than the same exercise without biofeedback. Therefore, for restoring the rotator cuff muscle balance in subjects with shoulder impingement syndrome, visual EMG biofeedback might augment established strengthening exercises (Reinold et al., 2004, 2009; Bitter et al., 2007; Ha et al., 2013). While a strengthening exercise can increase strength in general, without altering the motor control and abnormal muscle activation patterns, EMG-biofeedback-guided training specifically targets insufficient muscle activation and can potentially alter the motor control (Holtermann et al., 2010). Consequently, the side-lying shoulder external rotation exercise with visual EMG biofeedback is a promising clinical approach for restoring the rotator cuff muscle balance in subjects with shoulder impingement syndrome and shoulder instability. Furthermore, the sidelying shoulder external rotation exercise with visual EMG biofeedback can be incorporated in athletic conditioning programs for shoulder injury prevention and performance enhancement.

This study has several limitations. First, the current experiment did not test motor learning. The subjects may be successful in altering their motor performances while getting feedback but it does not mean that would continue to do so afterwards when tested for the same or similar task. Clinicians need to know if the decrease in EMG activity in posterior deltoid is also possible without visual EMG biofeedback. Therefore, further research is needed to examine the evaluation of retention. Second, the generalizability of our results is limited because subjects were asymptomatic and between 20–25 years of age. Further study should investigate the effects of biofeedback during the rotation exercise in subjects of all ages and patients with shoulder impingement. Third, EMG data were collected during the isometric contraction phase, and changes in the infraspinatus and posterior deltoid activity during dynamic contraction of the shoulder should be investigated. Fourth, kinematic data from the shoulder joint were not collected, so the contribution of changing posterior deltoid and infraspinatus activity cannot be confirmed. Finally, caution should be taken when interpreting these results because the long-term effects of using visual EMG biofeedback were not examined. In the future, a longitudinal study with kinematic measurements is required to determine the changes in the EMG amplitude of the posterior deltoid and infraspinatus as well as the infraspinatus/posterior deltoid EMG activity ratio.

In conclusions, the present study demonstrated that the visual EMG biofeedback significantly decreased posterior deltoid activity while increasing infraspinatus activity and the infraspinatus/posterior deltoid activity ratio during the side-lying shoulder external rotation exercise. Performing the side-lying shoulder external rotation exercise with biofeedback may be clinically helpful to prevent excessive posterior deltoid activation while inducing greater infraspinatus activation.

## Figures and Tables

**Table 1:** General characteristics of the subjects

Variables	Mean ± SD
Age (years)	21.5 ± 1.6
Body height (cm)	170.4 ± 7.9
Body mass (kg)	62.2 ± 10.8
BMI^a^ (kg/m^2^)	21.3 ± 3.0

BMI = body mass index
